# Aluminum Nitride-Based Adjustable Effective Electromechanical Coupling Coefficient Film Bulk Acoustic Resonator

**DOI:** 10.3390/mi14010157

**Published:** 2023-01-07

**Authors:** Yuanhang Qu, Tiancheng Luo, Zhiwei Wen, Min Wei, Xiyu Gu, Xiang Chen, Yang Zou, Yao Cai, Yan Liu, Chengliang Sun

**Affiliations:** 1 The Institute of Technological Sciences, Wuhan University, Wuhan 430072, China; 2 Key Laboratory of Artificial Micro, and Nano-Structures of Ministry of Education, School of Physics and Technology, Wuhan University, Wuhan 430072, China

**Keywords:** FBAR, adjustable effective electromechanical coupling coefficient, aluminum nitride

## Abstract

The arrival of the 5G era has promoted the need for filters of different bandwidths. Thin-film bulk acoustic resonators have become the mainstream product for applications due to their excellent performance. The *K_eff_*^2^ of the FBAR greatly influences the bandwidth of the filter. In this paper, we designed an AlN-based adjustable *K_eff_*^2^ FBAR by designing parallel capacitors around the active area of the resonator. The parallel capacitance is introduced through the support column structure, which is compatible with conventional FBAR processes. The effects of different support column widths on *K_eff_*^2^ were verified by finite element simulation and experimental fabrication. The measured results show that the designed FBAR with support columns can achieve a *K_eff_*^2^ value that is 25.9% adjustable.

## 1. Introduction

The advent of the 5G era has put forward higher requirements for RF communication technology: the frequency bands are more compact and the number of frequency bands has increased dramatically. Various bands have different bandwidth requirements, which means that filters with different bandwidths are increasingly required [1,2,3,4].

MEMS acoustic filters are a mainstream application in the RF field due to their small size and excellent performance [5,6]. As a representative product of acoustic filters, film bulk acoustic resonators (FBARs) are widely used owing to their high frequency, small size, and compatibility with Complementary Metal Oxide Semiconductor (CMOS) processes [7,8,9]. The FBAR is composed of a piezoelectric sandwich film above a cavity, and the frequency of the FBAR can be changed by adjusting the film thickness of each layer. The filters can be built by combining FBARs at different frequencies in series and parallel [10,11].

The effective electromechanical coupling coefficient (*K_eff_*^2^) of an FBAR is a vitally important parameter closely related to the bandwidth of the filter [12]. Generally, it is necessary to reasonably design the *K_eff_*^2^ of the FBAR device to meet the bandwidth requirements of different frequency bands. For FBAR device design, stack design and passive component introduction can affect the *K_eff_*^2^ of the FBAR. The study of Hao Zhang et al. shows that by changing the ratio of piezoelectric stacks, *K_eff_*^2^ was reduced from 6.9% to 5.0%; however, it is difficult to control the thickness of stacks precisely during the fabrication process [13]. The research of Paras Chawla et al. shows that the introduction of a capacitor can reduce the bandwidth of the filter, whereas the introduction of passive components affects the quality factor (Q-factor) of the device and increases the volume of the filter [14]. Another effective approach is to integrate capacitive structures with resonators using MEMS processes, and Pang et al. integrated FBARs with electrostatic MEMS actuators, achieving a tuning range of about 0.9% [15].

In this paper, we describe the design for an AlN-based FBAR with an adjustable *K_eff_*^2^, which can be tuned by paralleling capacitive structures around the active area of the resonator. The FBAR consists of a pentagon and five square support columns areas, and the top of the five support columns consists of a piezoelectric sandwich stack of Mo/AlN/Mo. The capacitor (that is connected to the FBAR in parallel) can be formed by the five additional sandwich stacks. The influence of squares with different areas on the *K_eff_*^2^ of the resonator was verified using the finite element method (FEM), and the designed devices were manufactured for further verification. The test results show that the width of the square ranges from 0 μm to 50 μm, and a parallel frequency adjustment range of 34 MHz at 2.56 GHz is realized.

## 2. Device’s Structural Design

**Figure 1:**
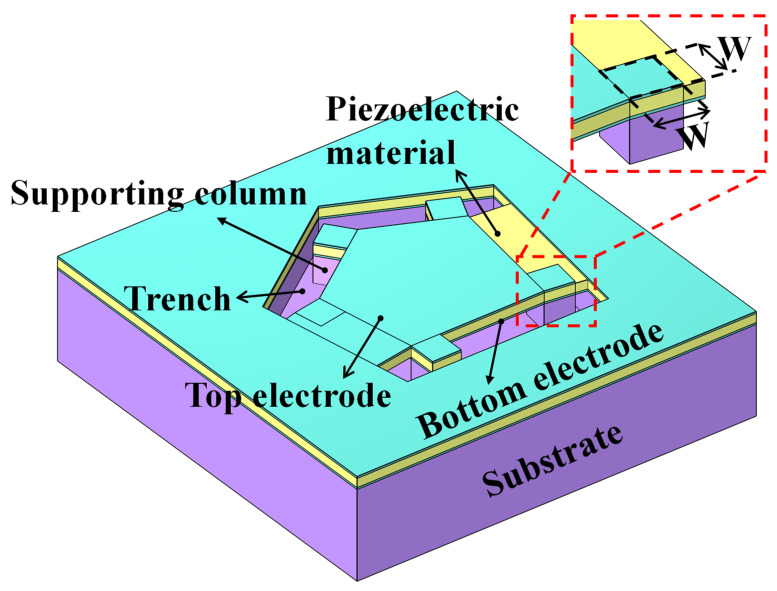
Sketch of the FBAR with supporting column structures.

The basic 3D configuration of the AlN-based adjustable *K_eff_*^2^ FBAR is shown in Figure 1. The active area of the resonator consists of a pentagonal area and five square areas. The five square areas are set at the five vertices of the pentagon, extending outward. A support column structure is placed under the five square areas to ensure the mechanical strength of the resonator. The width of the support column is defined as W. The groove structure is formed by etching the piezoelectric material around the active region of the resonator to reduce the acoustic energy leakage of the resonator and ensure the release of the sacrificial layer at the bottom of the resonator.

**Figure 2:**
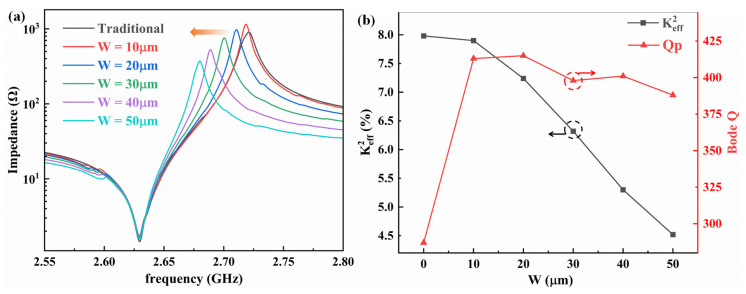
(**a**) The simulated impedance curves of the FBARs with support columns of different widths. (**b**) The performance dependence of FBARs with support columns of different widths.

Finite element method (FEM) simulation was performed in COMSOL Multiphysics to demonstrate the tuning of the *K_eff_*^2^. The piezoelectric stack consists of Mo/AlN/Mo with thicknesses of 200 nm/1000 nm/200 nm. The fixed constraint is imposed on the bottom of the substrate. The traditional FBAR (traditional pentagon structure) is also simulated as a control group. The material parameters used in the simulation are all COMSOL defaults. The impedance response curves obtained from the simulation are shown in Figure 2a. As W increases, the parallel frequency of the FBAR continues to decrease, while the series resonant frequency remains almost constant. As W increases from 0 µm to 50 µm, the parallel resonant frequency drops from 2.721 GHz to 2.682 GHz. The performance parameters of the FBAR were extracted through the impedance curve. For the longitudinal mode FBAR, the effective electromechanical coupling coefficient (*K_eff_*^2^) was calculated by Equation (1):

(1) $$K_{\mathrm{eff}}^{2}=\frac{\pi^{2}}{4}\frac{f_{s}}{f_{p}}\frac{f_{p}-f_{s}}{f_{p}}$$

where *f_s_* and *f_p_* are series and parallel resonant frequencies. The Q-factor is estimated from the Bode method with an *S*_11_ group delay and is calculated by Equations (2) and (3).

(2) $$Q_{\mathrm{Bode}}=\omega \times \frac{\left|S_{11}\right|\text{group\_delay}(S_{11})}{1-{\left|S_{11}\right|}^{2}}$$

(3) $$Q_{s,p}=\frac{f_{s,p}}{2}\left|\frac{d\Phi }{df}\right|$$

The performance dependence of FBARs with support columns of different widths is shown in Figure 2b. It can be clearly seen that the *K_eff_*^2^ value gradually decreases with the increase in column width. The Q-factor of FBARs with support columns of different widths is better than that of traditionally structured FBARs.

The modified Butterworth–Van Dyke (MBVD) fitting model is used to explain the variation in parallel resonant frequency. *C*_0_, *C_m_*, *L_m_*, *R_m_*, *R_s_*, and *R*_0_ are static capacitance, motional capacitance, motional inductance, motional resistance, electrode ohmic loss parasitic, and acoustic loss parasitic, respectively [16]. The calculations used to obtain *f_s_* and *f_p_* are as follows [17]:

(4) $$f_{s}=\frac{1}{2\pi \sqrt{L_{m}C_{m}}}$$

(5) $$f_{p}=\frac{1}{2\pi }\sqrt{\frac{C_{m}{+C}_{0}}{C_{m}C_{0}L_{m}}}{=f}_{s}\sqrt{\frac{C_{m}}{C_{0}}+1}$$

Compared to the conventional FBAR, the five additional square stack regions result in a shift in the parallel resonant frequency of the FBAR with support columns. In the static case, the FBAR can be seen as a parallel-plate capacitor whose dielectric is a piezoelectric material. The five additional square areas are equivalent to five capacitors in parallel around the FBAR. The capacitor capacitance value (*C_p_*) is related to the distance and area between the plates, which can be calculated by Equation (6):

(6) $$C_{p}=\frac{{5*S}_{\mathrm{square}}}{S_{\mathrm{pentagon}}}{\times C}_{0}$$

where *C*_0_ is the static capacitance extracted from the traditional FBAR MBVD model, *S_square_* is the area of a square, and *S_pentagon_* is the area of the pentagon. When the resonator is paralleled with a capacitor, through the MBVD model, *f_p_* can be expressed as [18]:

(7) $$f_{p}=\frac{1}{2\pi }\sqrt{\frac{C_{m}{+C}_{0}{+C}_{p}}{C_{m}L_{m}{(C}_{0}{+C}_{p})}}{=f}_{s}\sqrt{\frac{C_{m}}{C_{0}{+C}_{p}}+1}$$

**Figure 3:**
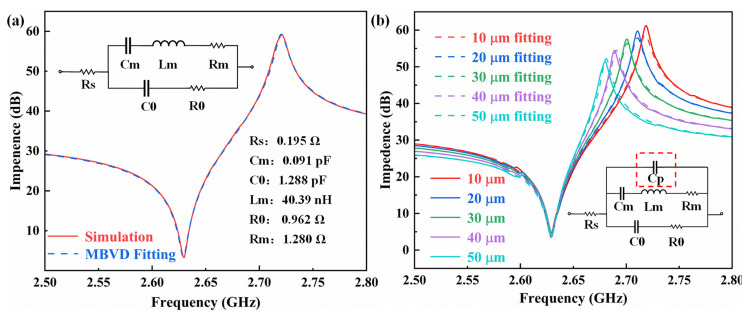
(**a**) Simulated impedance curve of a traditional FBAR and MBVD model fitting curve. (**b**) Simulated impedance curves of FBARs with support columns of different widths and parallel capacitor MBVD fitting curves.

To prove the effect of the support column on capacitance, the MBVD model of the traditional FBAR is extracted, and the extracted parameters are shown in Figure 3a. We then calculated the parallel capacitance of FBARs with support columns of different widths according to Equation (4). The calculated capacitance values are listed in Table 1. The capacitance increases gradually with the increase in column width. The width of 0 μm represents a FBAR without support columns.

We connected the different calculated capacitors in parallel to the MBVD model of the traditional FBAR, and the obtained frequency response curves are shown in the Figure 3b. The simulation curves for support columns of different widths are represented by solid lines, and the MBVD parallel capacitor fitting curves are represented by dashed lines. The results show that the shunt capacitor MBVD model can fit the impedance curves of support columns of different widths well.

## 3. Device Fabrication and Experiment

**Figure 4:**
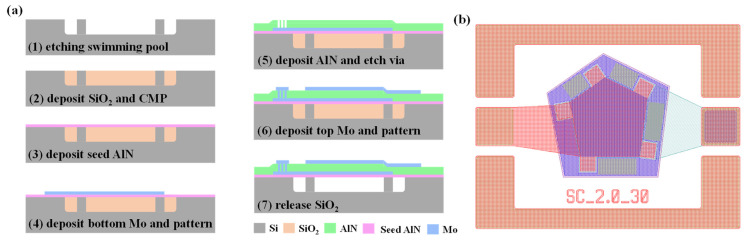
(**a**) Flow diagram of the fabrication process for the device. (**b**) The layout of the designed device.

The designed FBAR devices were fabricated to verify the simulation results. The fabrication process is illustrated in Figure 4a. We first etched a 2.5 μm swimming pool on a blank silicon wafer via deep reactive ion etching (DRIE). The pattern of the swimming pool was designed to form support columns during DRIE. SiO_2_ was used as a sacrificial layer and was deposited by low-pressure chemical vapor deposition (LPCVD). The superfluous SiO_2_ was removed by chemical mechanical polishing (CMP). A 25 nm thick AlN seed layer was deposited by magnetron sputtering to guarantee the crystal quality of the piezoelectric layer. Next, the bottom Mo electrode was deposited and patterned. The AlN piezoelectric layer was then deposited and formed by inductively coupled plasma (ICP) etching to ensure bottom electrode connectivity. After that, the top electrode was deposited and patterned. Finally, the SiO_2_ of the sacrificial layer was released by wet etching. The layout of the designed device is shown in Figure 4b.

**Figure 5:**
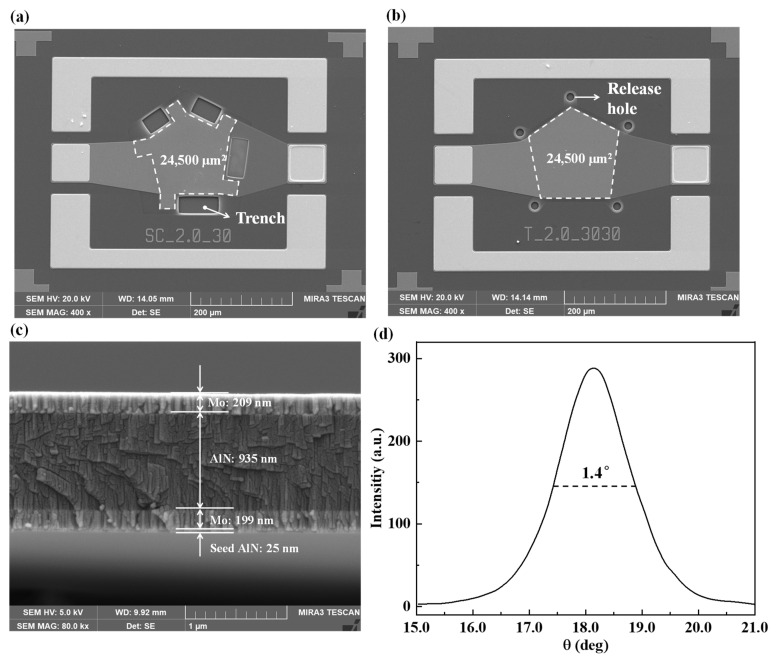
(**a**) SEM image of the fabricated FBAR with support columns. (**b**) SEM image of the traditional FBAR. (**c**) Cross-sectional SEM image of the piezoelectric stack. (**d**) The measured XRD rocking curve of the AlN layer.

SEM images of the fabricated FBAR with support columns are shown in Figure 5a, with the active area of the resonator outlined by a white dashed line and marked in the figure. The trench is used to release the sacrificial layer. Traditional pentagonal FBARs were also fabricated to serve as controls. A SEM image of the traditional FBAR is shown in Figure 5b. Unlike the FBAR with support columns, the traditional FBAR was released through release holes. As is shown in Figure 5c, the piezoelectric stack consists of Mo/AlN/Mo at thicknesses of 199 nm/935 nm/209 nm, and a 25 nm thick AlN seed layer is under the bottom Mo layer. The full-width half-maximum (FWHM) value of the rocking curve of the (0002) peak is shown in Figure 5d.

**Figure 6:**
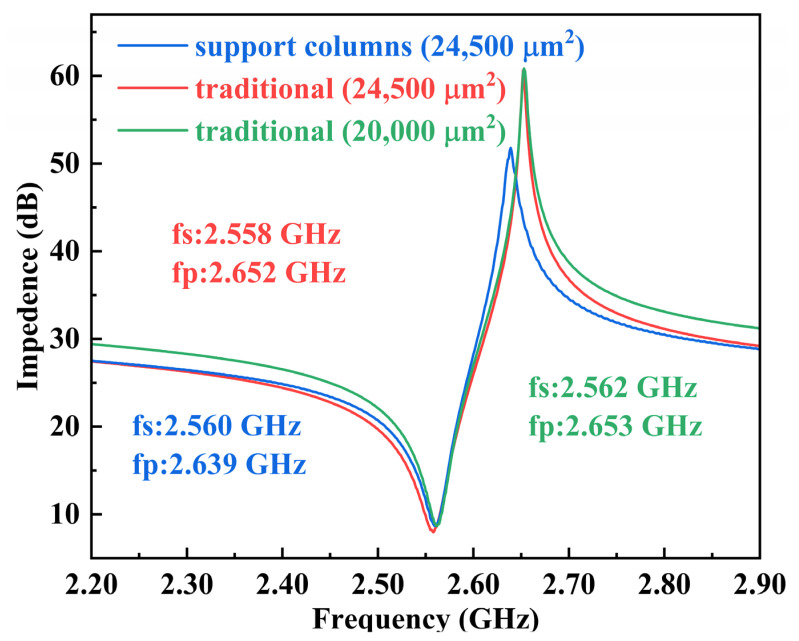
The measured results of different FBARs.

In general, changes in active area do not change the resonant frequency of the resonator. However, the increased active area due to the introduction of the support columns results in a decrease in the parallel frequency. To exclude the influence of area, we compared three FBAR devices with different structures: a 24,500 μm^2^ FBAR with support columns (support column width of 30 μm), a 24,500 μm^2^ conventional pentagonal FBAR, and a 20,000 μm^2^ conventional pentagonal FBAR. The devices were measured via wafer probing with a Keysight Network Analyzer (N5222B) connected to a Cascade Microtech GSG probe station. The comparison result is shown in Figure 6. Compared to the traditional pentagonal FBARs with different areas, the resonant frequency remains basically unchanged. However, when comparing the FBAR with support columns to a traditional pentagonal FBAR of the same area, the series resonance frequency of the resonator remains basically unchanged, though the parallel resonance frequency does shift. According to the above results, we can exclude the effect of area on the resonator, and the influence of the parallel capacitance brought by the support column can be confirmed.

**Figure 7:**
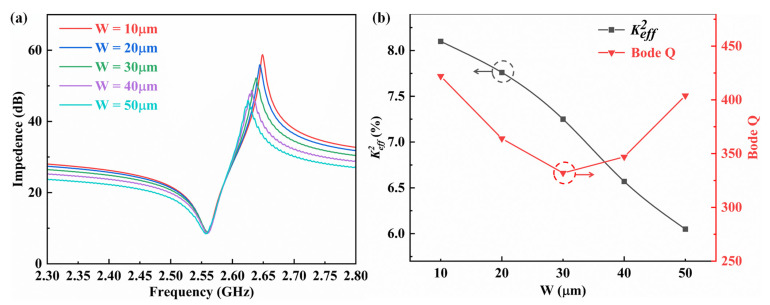
(**a**) The test results of the FBARs with support columns of different widths. (**b**) The measured performance dependence of FBARs with support columns of different widths.

The test results of FBARs with support columns of different widths are shown in Figure 7a. The performance parameters extracted from the measured impedance curves are shown in Figure 7b. As the width of the support column increases, the parallel resonant frequency of the resonator gradually decreases and the series resonant frequency remains basically unchanged, which is consistent with our simulation results. From the extracted performance parameter results, it can be seen that as the width of the support column increases from 10 μm to 50 μm, the measured *K_eff_*^2^ gradually decreases from 8.1% to 6.0%, achieving a *K_eff_*^2^ value that can be adjusted by up to 25.9%. As support column width increases from 10 μm to 30 μm, Q-factor gradually decreases. As support column width increases from 30 μm to 50 μm, Q-factor gradually increases. A comparison to other published device results is shown in Table 2 and demonstrates the clear advantages of the proposed devices.

**Figure 8:**
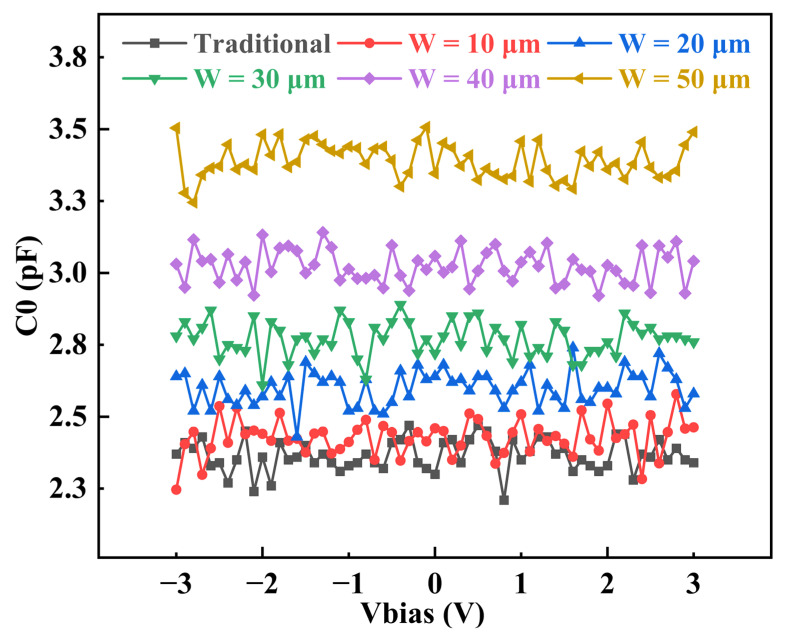
The measured C-V curves of the FBARs with support columns of different widths.

The C-V curves of different devices were measured by a semiconductor parameter analyzer (B1500A). The measured results are shown in Figure 8. The measured results indicate that capacitance increases gradually as the width of the support column increases. Parallel capacitors are equivalent to the superposition of capacitors. The parallel capacitance increases with increasing support column width, and the increase in parallel capacitance leads to an increase in resonator capacitance.

## 4. Conclusions

In this work, we designed an AlN-based adjustable *K_eff_*^2^ FBAR to enable *K_eff_*^2^ adjustment by introducing a shunt capacitance made possible by designing a support column structure around the active area of the resonator. The influence of the support column on the FBAR was verified using the finite element method, and the *K_eff_*^2^ adjustment mechanism was studied. We also theoretically proposed a calculation formula for parallel capacitance and verified it through simulation. The FBARs with support columns of different widths were fabricated to verify the simulation results. The test results show that the parallel resonant frequency and *K_eff_*^2^ decrease with increases in support column width, and the series resonant frequency basically remains unchanged. As support column width increases from 10 μm to 50 μm, a *K_eff_*^2^ adjustment range of 25.9% is achieved.

## Figures and Tables

**Table 1:** Calculated capacitance with support columns of different widths.

Width	0 μm	10 μm	20 μm	30 μm	40 μm	50 μm
*C_p_* (pF)	1.288	0.043	0.161	0.363	0.644	1.066

**Table 2:** Comparison to other published device results.

Piezoelectric Material	Freq (GHz)	Δf (MHz)	Δf/Freq	Ref.
ZnO	2.30	18	0.78%	[19]
ZnO	2.80	26	0.93%	[15]
BST	5.01	12	0.24%	[20]
Sc_0.2_Al_0.8_N	5.25	43	0.82%	[18]
AlN	2.56	24	0.94%	This work

## Data Availability

Data and code are available from the corresponding authors upon reasonable request.
